# A new method for reduction of incarcerated gravid uterus

**DOI:** 10.1016/j.eurox.2023.100201

**Published:** 2023-06-03

**Authors:** Masaya Tanimura, Asuka Sakiyama, Shinya Yoshioka

**Affiliations:** Department of Obstetrics and Gynecology, Toyooka Hospital, Toyooka, Japan; Department of Obstetrics and Gynecology, Kobe City Medical Center General Hospital, Kobe, Japan; Department of Obstetrics and Gynecology, Yokohama General Hospital, Yokohama, Japan; Department of Obstetrics and Gynecology, Kobe City Medical Center General Hospital, Kobe, Japan; Department of Obstetrics and Gynecology, Ohkubo Hospital, Akashi, Japan

Dear Editor,

Incarceration of the gravid uterus is a rare condition (1/3000 pregnancies) [1]. Herein, we present the case of an incarcerated gravid uterus with a myoma that was successfully reduced using a new technique for manual reduction.

A 34-year-old woman (gravida 1, para 0) became pregnant following treatment at our hospital. She was diagnosed with a uterine myoma measuring 12 cm in diameter. She was referred to our hospital at the 8th week of gestation with constipation and lower abdominal pressure. The uterine cervix was difficult to identify on vaginal examination, and a giant myoma in the uterine fundus trapped in the Douglas pouch was suspected to have caused the retroverted uterus. Both MRI and ultrasound evaluations showed an elongated cervix displaced behind the symphysis pubis and an incarcerated gravid uterus with a degenerated myoma. At 16 weeks of gestation, manual reduction was performed under general anaesthesia with propofol and sevoflurane, and adequate muscle relaxation with rocuronium bromide. The patient was placed in a tilted lithotomy position. First, we attempted manual reduction with cephalad digital pressure during pelvic examination, however, it did not work. Next, we inserted an ultrasound probe into the posterior fornix of the vagina and applied pressure to the myoma at the Douglas pouch, however, the myoma did not move. Finally, we tried to use the operator’s fist instead of an ultrasound probe, and simultaneously, his assistant placed both hands over the abdominal wall and attempted to lift the uterus and myoma ventrally and cephalad (Fig. 1). Incarceration was relieved, and the uterine cervix became palpable. After manual reduction, ritodrine hydrochloride was administered intravenously to suppress the uterine contractions. The patient complained of abdominal pain which was managed using analgesics. Incarceration of the gravid uterus did not recur, and the patient delivered vaginally at 40 weeks of gestation without any problems. The newborn girl weighed 3360 g.

**Fig. 1:**
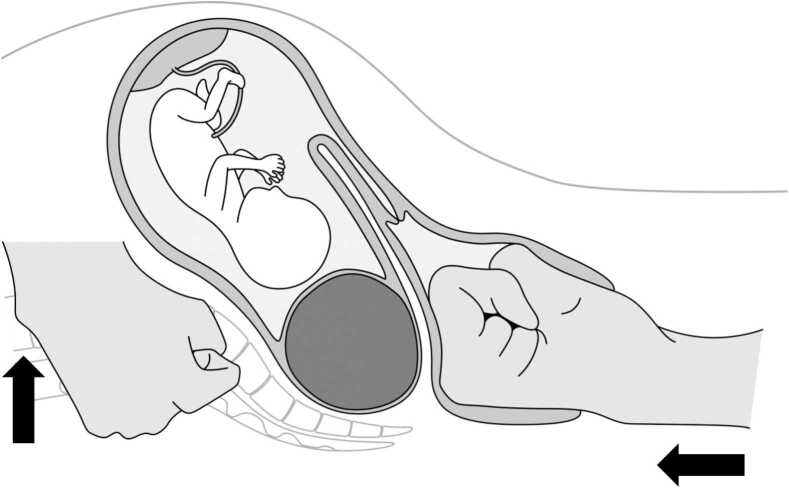
A new technique for manual reduction. Operator’s fist in vagina presses the uterine fundus while the assistant’s hands over the lateral region of the abdomen lifts the uterus and myoma ventrally and cephalad.

In the present case, we believe that the following two points were key to success: In the transvaginal manipulation, only the direction of the force was cephalad. The myoma was locked in the Douglas pouch and blocked with the sacral promontory if cephalad pressure was applied vaginally. Therefore, we applied a force to hold and lift the uterus ventrally with both hands through the abdominal wall. This bilateral abdominal compression significantly contributed to repositioning. By lifting the uterus ventrally, a space is created in the abdominal cavity, and the uterus is easily repositioned. This combination of transvaginal and transabdominal manipulations may be less invasive to the uterus and foetus by distributing the force on the uterus. However, this technique has not yet been reported in the literature. Another limitation is that manual reduction was performed under general anaesthesia with adequate analgesia and muscle relaxation. Relaxation of the rectus abdominis is important in the abdominal approach during this procedure. No complications were attributed to the use of analgesics or muscle relaxants in this case.

An incarcerated uterus can cause preterm labour, uterine rupture, urinary dysfunction, intrauterine foetal death, and foetal growth restriction [2]. In cases with failed simple manual manoeuvre for incarcerated uterus colonoscopic insufflation of the rectosigmoid, laparoscopy and laparotomy has been reported [1], [2]. Colonoscopy carries a risk of trauma and requires proficiency in bowel preparation. Surgical procedures can lead to severe maternal and foetal morbidities and longer recovery time [2]. This new technique makes it easier, and less invasive.

In conclusion, we report the case of an incarcerated gravid uterus with a myoma that was successfully repositioned using a new technique combining transabdominal and transvaginal manipulation.

## Funding statement

No funding was received for this research.

## Declaration of Competing Interest

The authors declare that they have no known competing financial interests or personal relationships that could have appeared to influence the work reported in this paper.
